# Rickettsial Infections Causing Acute Febrile Illness in Urban Slums, Brazil

**DOI:** 10.3201/eid2810.220497

**Published:** 2022-10

**Authors:** John B. Fournier, Lucas S. Blanton, Nivison Nery, Elsio A. Wunder, Federico Costa, Mitermayer G. Reis, Guilherme S. Ribeiro, David H. Walker, Albert I. Ko

**Affiliations:** Yale School of Public Health, New Haven, Connecticut, USA (J.B. Fournier, E.A. Wunder Jr., F. Costa, M.G. Reis, A.I. Ko);; University of Texas Medical Branch, Galveston, Texas, USA (L.S. Blanton, D.H. Walker);; Fundação Oswaldo Cruz, Salvador, Brazil (N. Nery Jr., F. Costa, M.G. Reis, G.S. Ribeiro, A.I. Ko);; Universidade Federal da Bahia, Salvador (N. Nery Jr., F. Costa, M.G. Reis, G.S. Ribeiro)

**Keywords:** Rickettsia, bacteria, parasites, vector-borne infections, zoonoses, spotted fever group rickettsiae, typhus group rickettsiae, acute febrile illness, urban slums, Brazil

## Abstract

We conducted enhanced acute febrile illness surveillance in an urban slum community in Salvador, Brazil. We found that rickettsial infection accounted for 3.5% of urgent care visits for acute fever. Our results suggest that rickettsiae might be an underrecognized, treatable cause of acute febrile illness in impoverished urban populations in Brazil.

*Rickettsia* spp. are small, obligately intracellular, gram-negative bacilli. The genus includes the spotted fever group rickettsiae (SFGR) and typhus group rickettsiae (TGR). SFGR and TGR are underrecognized causes of acute febrile illness (AFI) worldwide, particularly in the tropics, because clinical manifestations of rickettsial infections are often indistinguishable from those of other endemic infections (*1*). Recent studies have suggested that SFGR profiles in Brazil might be shifting toward an increasing number of cases in urban areas (*2*). However, knowledge of whether rickettsiae cause AFI in urban settings is limited, especially in informal settlements where social determinants and the presence of potential vectors and reservoirs might favor transmission. Rickettsiae-mediated AFI has considerable public health and therapeutic implications because prompt administration of targeted antimicrobial therapy can reduce illness and death associated with rickettsioses (*3*).

To evaluate whether rickettsial infections might be a potential cause of AFI in an urban slum setting, we performed enhanced surveillance from April 1, 2009, through October 31, 2012, in a public sector urgent care facility that served a community of ≈55,000 inhabitants in Salvador, Brazil (*4*). We identified and enrolled eligible outpatients who were >5 years of age and had a measured (>37.8°C) or reported fever of <21 days duration. We collected blood samples during the acute phase at the time of enrollment and during the convalescent phase >15 days later. We screened serum samples at an initial dilution of 1:64 using a rickettsial immunofluorescence assay in which the *Rickettsia rickettsii* Sheila Smith strain was used to determine SFGR IgG reactivity, and *R. typhi* Wilmington strain was used to determine TGR IgG reactivity. Laboratory-confirmed rickettsial infections were defined as seroconversion (negative acute-phase titer and convalescent-phase titer of >128) or a 4-fold increase in titer between acute- and convalescent-phase samples. We detected dengue viral infections using DENV reverse transcription PCR, Panbio Dengue Early ELISA (NS1 antigen-capture), and Bioline Dengue IgM-capture ELISA (Abbott Laboratories, https://www.abbott.com) (*5**,**6*) and leptospirosis using the microscopic agglutination test (*7*). The study was approved by the Oswaldo Cruz Foundation Committee on Ethics in Research, Brazilian National Council for Ethics in Research, and Yale University Institutional Review Board.

Among 5,035 enrolled patients with AFI, 1,016 (20.2%, 95% CI 19.1%–21.3%) had confirmed dengue and 137 (2.7%, 95% CI 2.3%–3.2%) had confirmed leptospirosis. Among the 3,882 patients who did not have dengue or leptospirosis, 1,016 (26.2%) did not fulfill criteria for an influenza-like illness (temperature >37.8°C, reported fever and cough, or sore throat for <7 days), and these patients provided acute- and convalescent-phase serum samples. We used a random number generator to select 200 patients from the 1,016 participants whose samples were evaluated in the rickettsial immunofluorescence assay. Of those 200 patients, we identified 6 (3.0%, 95% CI 1.4%–6.4%) patients who had SFGR-positive serum samples and 1 (0.5%, 95% CI 0.0%–2.8%) patient who had a TGR-positive serum sample (Table).

**Table:** Clinical characteristics of 7 patients and IFA serum titers for SFGR or TGR IgG reactivity in study of rickettsial infections causing acute febrile illness in urban slums, Brazil*

Patient no.	Age, y/sex	Symptoms	Duration of illness, d†	Antigen (group)	IFA titers	
					Acute phase‡	Convalescent phase§
1	10/F	Fever, headache, myalgia	6	*R. rickettsii* (SFGR)	0	256
2	21/F	Fever, headache, myalgia	1	*R. rickettsii* (SFGR)	0	256
3	42/F	Fever, headache, myalgia, lethargy, diarrhea	3	*R. rickettsii* (SFGR)	0	256
4	6/M	Fever, headache, myalgia, lethargy, vomiting, diarrhea	10	*R. rickettsii* (SFGR)	0	128
5	8/F	Fever, headache, myalgia, fatigue, vomiting, diarrhea	6	*R. rickettsii* (SFGR)	0	256
6	12/M	Fever, headache	1	*R. rickettsii* (SFGR)	128	512
7	6/F	Fever, lethargy, diarrhea	5	*R. typhi* (TGR)	0	256

*Rickettsia rickettsii Sheila Smith strain was used to determine SFGR IgG reactivity and *R. typhi* Wilmington strain was used to determine TGR IgG reactivity. IFA, immunofluorescence assay; SFGR, spotted fever group rickettsiae; TGR, typhus group rickettsiae. †Number of days of illness before attending urgent care facility in Salvador, Brazil. ‡Acute-phase serum samples were obtained at the time of enrollment when the patient arrived initially at the urgent care facility. §Convalescent-phase serum samples were obtained >15 d after enrollment in the study.

Patients who had SFGR- and TGR-positive samples were 6–42 years of age, and 5 of the 7 patients were women. All 7 patients had measured (>37.8°C) or reported fever, 6 of 7 patients reported headaches, and 5 of 7 patients reported myalgia. Patients did not report a rash (Table). The 7 patients attended the urgent care facility 1–10 days after the onset of symptoms and had mild self-limiting illnesses. Those patients did not receive antimicrobial therapy or require hospitalization; symptoms resolved within 30 days of onset.

Identifying rickettsial infections among patients attending an urgent care facility in an urban center in Brazil is noteworthy because rickettsiae have not previously been described as a substantial cause of AFI in urban populations in this country. Of note, the immunofluorescence assay used is unable to differentiate among *Rickettsia* spp. *R. rickettsii* is a well-described cause of spotted fever in Brazil in rural settings, and *R. parkeri* is an emerging cause of infection (*8*–*10*). Antibodies that are cross-reactive with *R. rickettsii* can be stimulated by *R. parkeri*, *R. akari*, and other SFGR. *R. typhi* has been rarely reported as a cause of AFI in urban settings. Other rickettsial species identified in Brazil are *R. felis*, *R. rhipicephali*, *R. bellii*, *R. amblyommatis*, *R. andeanae*, and *R. monteiroi*, although their pathogenicity is unclear (*10*).

Although all causative rickettsial species, potential vectors, and reservoirs have yet to be identified, this study suggests that rickettsiae might be a cause of AFI in urban slum settings in Brazil. A limitation of this study is that it was performed in a single urban center; further studies will be needed to confirm the generalizability of these findings. However, these findings raise clinical awareness for rickettsiae as a potential cause of AFI in urban slum populations in the tropics and the possible need for empiric antimicrobial therapy in suspected cases, especially because diagnostic testing is often lacking in these urban environments.
